# Complement Factor H Mutation-Associated Atypical Haemolytic Uraemic Syndrome Presenting With Dialysis-Dependent Renal Failure: A Case Report

**DOI:** 10.7759/cureus.117074

**Published:** 2026-09-28

**Authors:** Anil Jain, Deyab Mohamed, Gauri Satheesh Nair, Marios Magriplis, Ahmed Yusuf, Gabrielle Matthews

**Affiliations:** 1 Nephrology, United Lincolnshire Teaching Hospitals NHS Trust, Lincoln, GBR; 2 Medicine, United Lincolnshire Teaching Hospitals NHS Trust, Lincoln, GBR; 3 Medicine, Lincoln County Hospital, United Lincolnshire Teaching Hospitals NHS Trust, Lincoln, GBR; 4 Pathology, University Hospitals of Leicester NHS Trust, Leicester, GBR

**Keywords:** acute kidney failure, atypical haemolytic uraemic syndrome, complement factor h mutation, complement-mediated thrombotic microangiopathy, dialysis-dependent, genetic testing, haemolytic anaemia, kidney biopsy

## Abstract

A 57-year-old gentleman with no prior kidney disease presented with rapidly progressive acute kidney injury, anaemia, and hypertension. Initial investigations revealed severe acute kidney injury, microangiopathic haemolysis, and heavy proteinuria. Secondary causes of thrombotic microangiopathy (TMA), including Shiga toxin infection, autoimmune disease, malignant hypertension, and medication-induced injury, were systematically excluded. A renal biopsy demonstrated morphological features of TMA and helped exclude important mimics. Subsequent genetic testing identified a pathogenic complement factor H (CFH) splice site mutation, supporting a diagnosis of complement-mediated atypical haemolytic uraemic syndrome (aHUS). He required urgent dialysis. Following counselling and meningococcal vaccination, complement inhibition with eculizumab was offered, but the patient declined because of concerns about lifelong infection risk. He remains dialysis dependent.

This case highlights the role of kidney biopsy in characterising injury and excluding mimics, the importance of recognising complement-mediated disease early, and the need for thoughtful, shared decision-making when treatment involves long-term immunological risk.

## Introduction

Atypical haemolytic uraemic syndrome (aHUS) is a rare cause of thrombotic microangiopathy (TMA), distinguished from typical (Shiga toxin-associated) HUS by the absence of a diarrheal prodrome and driven by uncontrolled activation of the alternative complement pathway [1-3].

Among the known genetic contributors, complement factor H (CFH) variants account for approximately 20%-30% of aHUS and often carry a poorer renal prognosis [4,5]. CFH normally regulates the alternative complement pathway by limiting complement activation on host cell surfaces; loss or dysfunction of CFH-mediated regulation can result in uncontrolled complement activation, endothelial injury, and subsequent TMA. Although complement blockade has transformed outcomes [6,7], it requires lifelong therapy and heightened infection vigilance.

This case illustrates the real-world complexity of diagnosing complement-mediated disease and navigating treatment decisions that carry long-term consequences for patients.

## Case presentation

A 57-year-old gentleman with a history of hypertension (no prior kidney disease; baseline creatinine approximately 100 μmol/L) presented with several days of increasing leg swelling, facial puffiness, and progressive shortness of breath. He reported profound fatigue and very low urine output. He regularly used ibuprofen (400-600 mg daily) for musculoskeletal discomfort over several months. There was no diarrhoeal illness, fever, or bleeding.

On admission, he appeared clinically fluid overloaded, with blood pressure ranging from 153-190/97-110 mmHg, with no clinical signs of hypertensive emergency. Initial investigations demonstrated severe acute kidney injury, with a creatinine of 786 μmol/L (baseline 98 μmol/L six months earlier), eGFR 6 mL/min/1.73 m², and urea 29.1 mmol/L. Urine protein ratio was 611 mg/mmol, consistent with nephrotic-range proteinuria. Renal ultrasound showed normal-sized kidneys. He also had severe anaemia (haemoglobin 63 g/L), with schistocytes on the blood film, elevated lactate dehydrogenase (809 U/L), and markedly reduced haptoglobin (<0.10 g/L), consistent with microangiopathic haemolysis. Bilirubin was 18 μmol/L, while platelet counts remained relatively preserved at 140-157 ×10⁹/L, with no significant thrombocytopenia. Although severe thrombocytopenia is classically associated with thrombotic thrombocytopenic purpura (TTP), platelet count alone cannot reliably distinguish TTP from complement-mediated aHUS, as significant thrombocytopenia may also occur in aHUS. In this case, the relatively preserved platelet count, together with the predominant severe renal involvement, triggered further investigation.

By this stage, given the severe hypertension and evidence of microangiopathic haemolysis, hypertension-associated TMA was considered an important differential.

Infective screening was negative apart from respiratory polymerase chain reaction positivity for influenza A and respiratory syncytial virus (RSV). The concurrent influenza A and RSV infections were considered potential precipitating factors for complement-mediated aHUS. Viral infections can activate the complement system and may trigger TMA in genetically susceptible individuals. Shiga toxin testing was negative, while ADAMTS13 (a disintegrin and metalloproteinase with a thrombospondin type 1 motif, member 13) activity was 85.4 IU/dL, effectively excluding TTP.

Complement studies demonstrated reduced C3 (0.83 g/L) with normal C4 (0.37 g/L). Autoimmune screening, including antinuclear antibody (ANA), antineutrophil cytoplasmic antibody (ANCA), anti-glomerular basement membrane (anti-GBM), and anti-phospholipase A2 receptor (anti-PLA2R) antibodies, was negative. Given the diagnostic uncertainty, a renal biopsy was undertaken and demonstrated TMA with both acute and chronic features. Subsequent genetic testing identified a heterozygous CFH splice-site variant, c.3494-1G>T, with a normal CFH-related gene copy number. 

In this patient, the presence of a heterozygous CFH splice-site variant provided a potential underlying genetic predisposition, with the acute viral infections possibly acting as environmental triggers for disease onset. The underlying CFH splice-site variant could represent the genetic susceptibility (first hit), while the concurrent influenza A and RSV infections may have acted as environmental triggers (second hit), leading to complement activation and the clinical presentation of aHUS. Taken together, the clinicopathological and genetic findings favoured complement-mediated aHUS over hypertension-associated TMA or an event solely secondary to viral infection.

Important investigation findings

A comprehensive summary of clinical, laboratory, and genetic findings is presented in Table 1.

**Table 1:** Comprehensive summary of clinical, laboratory, and genetic findings is presented AKI: acute kidney injury; ADAMTS13: a disintegrin and metalloproteinase with a thrombospondin type 1 motif, member 13; ANA: antinuclear antibody; ANCA: antineutrophil cytoplasmic antibody; anti-GBM: anti-glomerular basement membrane antibody; anti-PLA2R: anti-phospholipase A2 receptor antibody; BP: blood pressure; CFH: complement factor H; CFHR: complement factor H-related; CFI: complement factor I; C3: complement component 3; C4: complement component 4; CFB: complement factor B; CD46: cluster of differentiation 46 (also known as membrane cofactor protein); DGKε: diacylglycerol kinase epsilon; eGFR: estimated glomerular filtration rate; HIV: human immunodeficiency virus; LDH: lactate dehydrogenase; MMACHC: methylmalonic aciduria and homocystinuria type C protein; PCR: protein:creatinine ratio; RSV: respiratory syncytial virus; STEC: Shiga toxin-producing *Escherichia coli*; TMA: thrombotic microangiopathy; TTP: thrombotic thrombocytopenic purpura

Parameter	Findings	Reference Range	Units	Interpretation
Demographics	57-year-old male	-	-	-
Clinical presentation	Leg swelling, facial puffiness, shortness of breath, oliguria; BP 153-190/97-110	BP <130/80	mmHg	AKI with fluid overload and severe hypertension
Serum creatinine	786 (baseline ~100)	62-115	μmol/L	Severe AKI
eGFR	6	≥60	mL/min/1.73 m²	Severe renal impairment
Serum urea	29.1	2.5-7.8	mmol/L	Severe uraemia
Haemoglobin	63	130-170	g/L	Severe anaemia
Blood film	Schistocytes present	No schistocytes	-	Microangiopathic haemolysis
LDH	809	135-225	U/L	Elevated, consistent with haemolysis
Haptoglobin	<0.10	0.30-2.00	g/L	Reduced, consistent with haemolysis
Bilirubin	18	<21	μmol/L	Within reference range - upper limit of normal
Platelets \	140-157	150-400	×10⁹/L	Mild/borderline thrombocytopenia
Urine protein:creatinine ratio	611	<15	mg/mmol	Nephrotic-range proteinuria
Complement C3	0.83	0.90-1.80	g/L	Reduced; suggests complement pathway activation
Complement C4	0.37	0.16-0.47	g/L	Normal
Infectious screen	Hepatitis B/C and HIV negative; influenza A and RSV positive; STEC negative	Negative	-	No evidence of infectious secondary TMA
ADAMTS13 activity	85.4	40-130	IU/dL	Normal; TTP unlikely
Autoimmune screen	ANA, ANCA, anti-GBM and anti-PLA2R negative	Negative	-	No evidence of autoimmune disease
Renal ultrasound	Normal-sized kidneys	-	-	No evidence of obstructive nephropathy
Renal biopsy (20/12/2024)	Glomerular capillary wall thickening, endothelial swelling and fibrin thrombi	-	-	Confirms thrombotic microangiopathy with acute and chronic features
Genetic testing (February 2025)	Heterozygous CFH splice-site variant c.3494-1G>T; normal CFHR1/2/3 copy number; no variants in CFI, CD46, C3, CFB, DGKε or MMACHC	No pathogenic variants	-	Supports complement-mediated aHUS

Renal biopsy findings supporting TMA

A renal biopsy performed on Day 11 of admission, before genetic testing results were available, demonstrated features consistent with TMA, prompting further evaluation for complement-mediated disease. The earliest diagnostic clue came from electron microscopy (Figure 1), which showed marked subendothelial widening filled with electron-lucent material, indicating acute endothelial injury. Light microscopy then further characterised the process, revealing glomerular capillary wall thickening, prominent endothelial swelling, fibrin thrombi, and areas suggestive of evolving chronic damage (Figure 2). These findings were supported by silver staining (Figure 3), which demonstrated double-contour formation of the GBM, a feature of chronic endothelial injury and glomerular remodelling. Additional vascular involvement was evident in Figure 4, which showed intimal mucoid oedema in a medium-sized artery, confirming that the TMA extended beyond the glomeruli into larger intrarenal vessels. Finally, immunoperoxidase staining (Figure 5) was negative, excluding an immune-complex-mediated process and supporting the diagnosis of non-immune-complex TMA with both acute and chronic pathological features.

**Figure 1:**
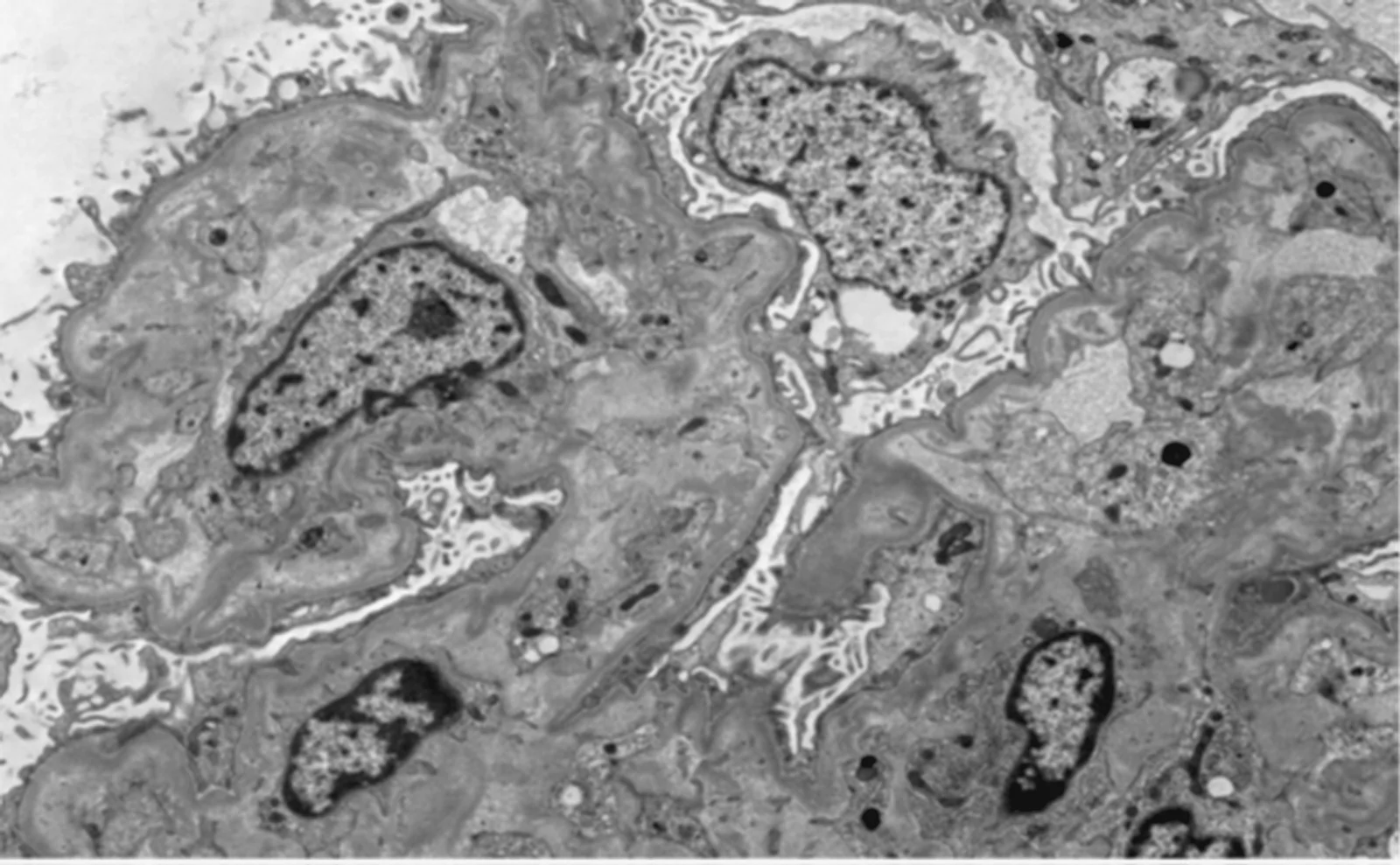
Electron microscopy: subendothelial widening with electron‑lucent material and foot process effacement

**Figure 2:**
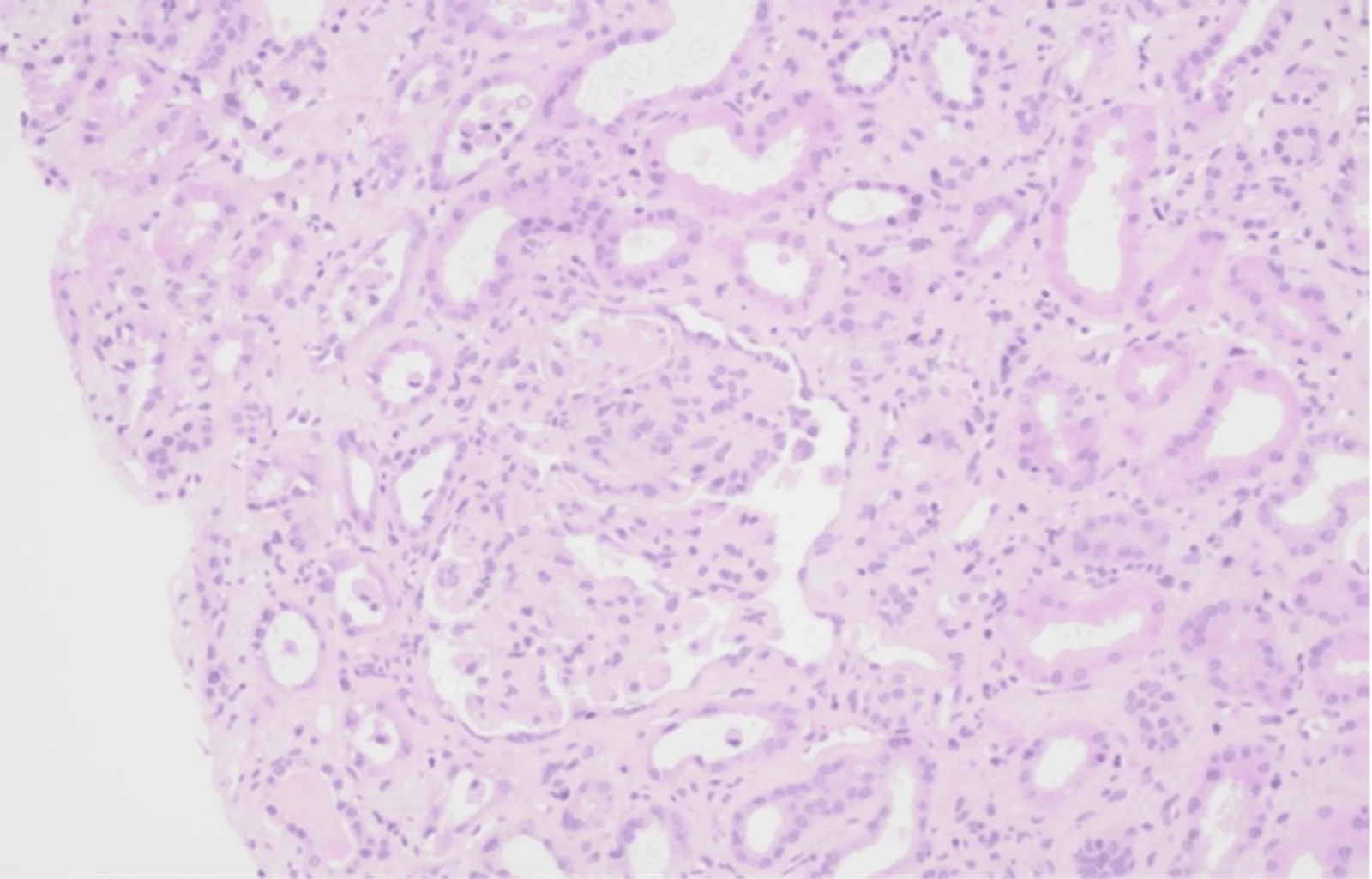
H&E stain: bloodless glomerulus with endothelial swelling

**Figure 3:**
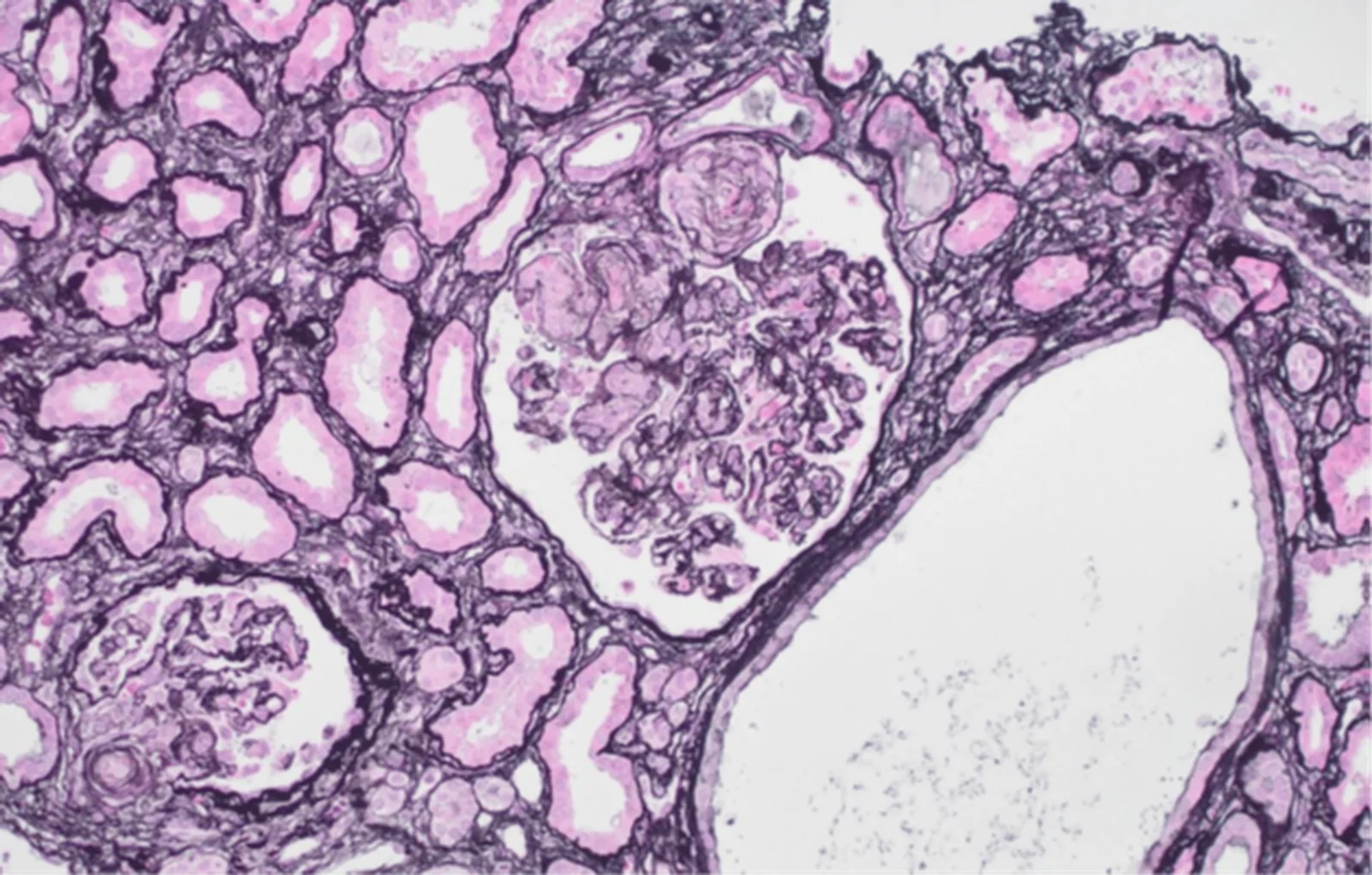
Silver stain: double‑contour formation of the glomerular basement membrane

**Figure 4:**
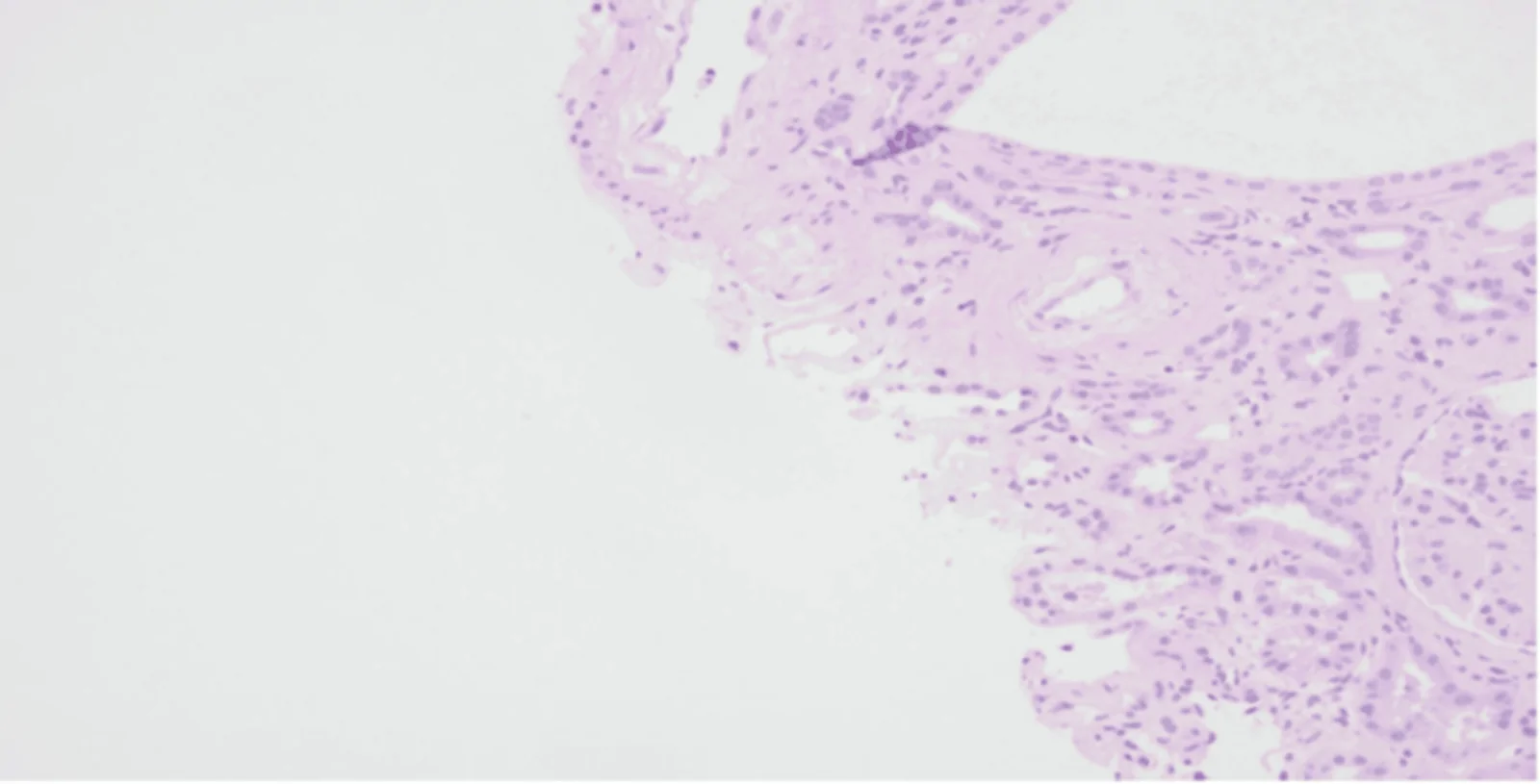
Vessel H&E: intimal mucoid oedema in a medium‑sized artery

**Figure 5:**
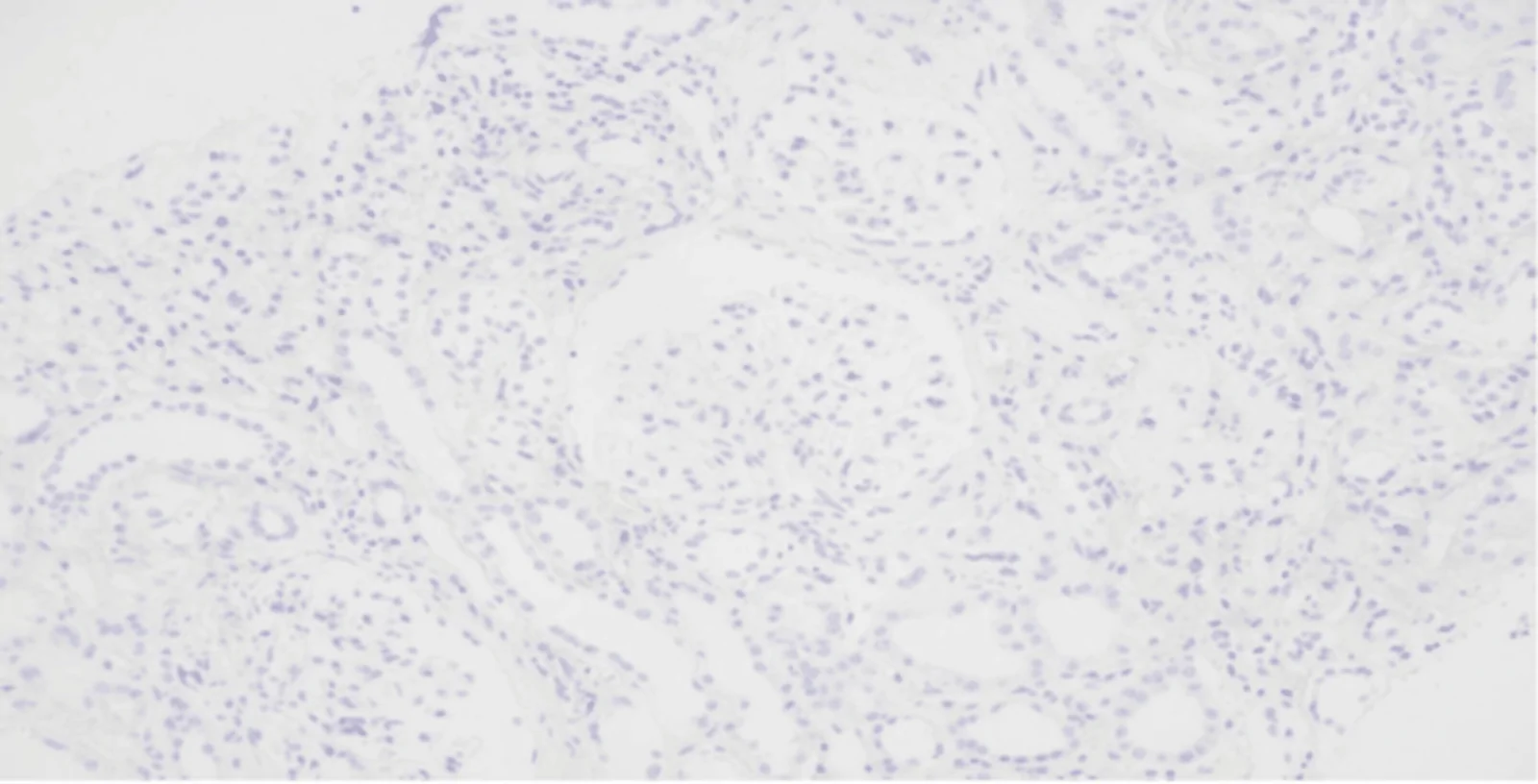
Immunoperoxidase panel: negative staining with no evidence of immune‑complex deposition

Differential diagnosis

The main diagnostic challenge was distinguishing complement-mediated TMA from other causes of TMA. TTP was considered initially; however, ADAMTS13 activity was normal (85.4 IU/dL), and platelet counts were relatively preserved, making TTP unlikely [8]. Shiga toxin-associated TMA was considered but was unlikely given the absence of a compatible gastrointestinal prodrome and negative stool Shiga toxin testing.

Secondary causes, including autoimmune disease, malignant hypertension, infection-related TMA, and medication-induced TMA, were excluded through clinical and laboratory assessment [8].

Secondary TMA due to severe hypertension was an important differential given the marked hypertension (up to 190/110 mmHg). However, the degree of renal injury, biopsy-proven TMA with chronic features, low C3, and subsequent identification of a CFH variant favoured an underlying complement-mediated process rather than hypertension as the primary cause. Infection-associated TMA was considered in view of concurrent influenza A and RSV infection. These infections may activate complement and potentially trigger TMA in genetically susceptible individuals; therefore, they were considered possible precipitating factors rather than an independent cause. Drug-induced TMA was considered given the history of nonsteroidal anti-inflammatory drug (NSAID) exposure, although the clinical and pathological findings did not establish a clear drug-induced mechanism. Autoimmune and other secondary causes were investigated, with negative ANA, ANCA, anti-GBM, and anti-PLA2R antibodies. Cobalamin C deficiency and diacylglycerol kinase epsilon (DGKE)-associated TMA were also excluded on subsequent genetic testing, with no variants identified in methylmalonic aciduria and homocystinuria type C protein (MMACHC) or DGKE.

Taken together, the severe renal involvement, low C3, biopsy-confirmed TMA, normal ADAMTS13 activity, exclusion of alternative causes, and identification of a heterozygous CFH splice-site variant supported a diagnosis of complement-mediated aHUS [9].

Treatment

He required immediate haemodialysis, initially via temporary access and later through a long-term catheter. Antihypertensive therapy and metabolic control were optimised.

After meningococcal vaccination, the patient was counselled regarding eculizumab, including its potential to promote renal recovery and reduce the likelihood of continued dialysis, while acknowledging that renal recovery could not be guaranteed given the severity and chronicity of his kidney injury. The risks of complement inhibition, particularly serious meningococcal and other encapsulated bacterial infections, and the need for ongoing vaccination and monitoring were discussed. The implications and treatment burden of long-term dialysis were also explained. Following discussion of these potential benefits and risks, the patient ultimately declined eculizumab due to his apprehension about immunosuppression and potential infection risks. He preferred to continue with dialysis.

Ravulizumab, a longer-acting C5 inhibitor requiring less frequent administration than eculizumab, represents an alternative treatment option for complement-mediated aHUS and may offer a reduced treatment burden for patients requiring long-term complement inhibition. However, ravulizumab was not discussed with the patient in this case. Complement inhibition with C5 inhibitors has been shown to improve renal outcomes and reduce progression to end-stage kidney disease (ESKD).

The decision to proceed with eculizumab was made following authorisation by the aHUS MDT team. No alternative treatment was offered in this case. The patient's decision to decline treatment was not related to the frequency of administration but rather to concerns regarding the potential infection risks associated with eculizumab therapy.

Plasma exchange was not indicated at presentation and was not proposed by the aHUS multidisciplinary team (MDT). Hence, it was not discussed with the patient.

Outcome and follow-up

At ongoing follow-up, the patient remains dialysis-dependent and continues to receive thrice-weekly haemodialysis, with stable blood pressure control. He is reviewed regularly at six- to eight-week intervals, during which complement blockade and its potential benefits and risks are revisited. The patient has made an informed decision to continue with dialysis rather than complement blockade. Follow-up focuses on dialysis support, cardiovascular risk management, and multidisciplinary renal care.

## Discussion

Complement-mediated aHUS arises from dysregulation of the alternative complement pathway, either due to genetic mutations (e.g., CFH, CFI, CD46, and C3) or, less commonly, acquired autoantibodies against CFH, leading to endothelial injury and microvascular thrombosis [1-3,9]. CFH mutations are the most commonly identified genetic abnormality and are associated with a higher risk of long-term kidney failure [4,5].

In this case, the renal biopsy was helpful in demonstrating morphological features consistent with TMA and in excluding alternative pathologies, such as immune complex-mediated disease or vasculitis, supported by negative immunoperoxidase staining (Figure 5). Drug-induced TMA was considered given the history of NSAID exposure, although biopsy cannot establish the underlying cause of TMA [8]. Biopsy showed TMA with both acute and chronic features: endothelial swelling, subendothelial widening, and fibrin thrombi (acute injury), alongside GBM double contours (chronic injury), with additional arterial involvement (intimal mucoid oedema).

The presence of a CFH variant suggests an underlying genetic susceptibility to complement-mediated aHUS. In keeping with the proposed two-hit hypothesis, the concurrent influenza A and RSV infections may have acted as environmental triggers for complement activation and precipitated clinical disease in this genetically susceptible patient. The identification of a CFH splice-site variant provided further diagnostic certainty and informed patient counselling.

Early initiation of complement inhibition has been shown to improve renal outcomes and survival, although the prospect of lifelong therapy and infection risk remains a major concern for many patients. Eculizumab, a humanised monoclonal antibody directed against complement protein C5, inhibits terminal complement activation and has dramatically improved renal survival in aHUS. Initiating treatment early reduces the likelihood of irreversible renal injury and progression to ESKD. However, long-term complement blockade poses an ongoing risk of serious infections, particularly due to *Neisseria meningitidis*, requiring vaccination and, in some cases, prophylactic antibiotics.

This case illustrates the complexity of clinical decision-making in rare diseases where effective treatments exist but carry substantial long-term implications. The patient's decision to decline eculizumab and remain dialysis-dependent carries its own long-term risks; population-based studies show that chronic kidney disease is a major contributor to sudden death in adults, accounting for over 25% of cases, primarily due to cardiovascular events [10].

Before eculizumab, CFH-associated aHUS had the most severe outcomes among all genotypes. In the Global aHUS Registry (851 patients), patients with CFH mutations had significantly reduced ESKD-free survival compared to other genotypes. Up to 50% of aHUS cases progressed to ESRF or developed irreversible brain damage, and 25% died during the acute phase before complement inhibition was available. Without complement inhibition, the prognosis of CFH mutation-associated aHUS is poor, with most patients progressing to ESKD within one year of presentation [11]. Recurrence following kidney transplantation is common, particularly with CFH mutations, where recurrence rates exceed 50% and often necessitate prophylactic eculizumab [9]. 

Looking ahead, next-generation complement inhibitors may offer improved safety profiles, longer dosing intervals, and reduced treatment burden. The development of next-generation complement inhibitors, such as ravulizumab, which has a comparable safety profile but improved dosing convenience [12], may in future offer alternative options for patients who decline conventional therapy.

Considered a genetic disease in which several mutation variants exist, incomplete penetrance within CFH variants, estimated at around 48%, suggests that carrying a pathogenic variant confers susceptibility rather than a guaranteed clinical phenotype of aHUS [13]. The two-hit hypothesis suggests that the clinical manifestations of aHUS follow an identified trigger, such as infection or drugs, in around 30%-50% of patients [14].

## Conclusions

Complement-mediated aHUS due to a CFH variant is a rare but severe cause of AKI and haemolysis. Renal biopsy plays a pivotal role in establishing TMA and assessing the extent and chronicity of renal injury, while genetic testing can support the diagnosis and provide important prognostic information regarding the risk of ESKD. This case highlights the importance of recognising complement-mediated TMA and considering targeted complement inhibition in appropriate patients.

Although complement inhibition with eculizumab remains the gold standard for treatment, patient autonomy and individual risk perception significantly influence management choices. This case highlights the need for early recognition, multidisciplinary discussion, and patient-centred decision-making in rare complement-mediated kidney diseases. The development of next-generation complement inhibitors with improved safety and dosing convenience may, in the future, offer alternative options for patients who decline conventional therapy.
